# DeepciRGO: functional prediction of circular RNAs through hierarchical deep neural networks using heterogeneous network features

**DOI:** 10.1186/s12859-020-03748-3

**Published:** 2020-11-12

**Authors:** Lei Deng, Wei Lin, Jiacheng Wang, Jingpu Zhang

**Affiliations:** 1 School of Computer Science and Engineering, Central South University, Changsha, 410075 China; 2 School of Computer and Data Science, Henan University of Urban Construction, Pingdingshan, 467000 China

**Keywords:** Gene ontology, Representation learning, HIN2Vec, Multi-label hierarchical classification

## Abstract

**Background:**

Circular RNAs (circRNAs) are special noncoding RNA molecules with closed loop structures. Compared with the traditional linear RNA, circRNA is more stable and not easily degraded. Many studies have shown that circRNAs are involved in the regulation of various diseases and cancers. Determining the functions of circRNAs in mammalian cells is of great significance for revealing their mechanism of action in physiological and pathological processes, diagnosis and treatment of diseases. However, determining the functions of circRNAs on a large scale is a challenging task because of the high experimental costs.

**Results:**

In this paper, we present a hierarchical deep learning model, DeepciRGO, which can effectively predict gene ontology functions of circRNAs. We build a heterogeneous network containing circRNA co-expressions, protein–protein interactions and protein–circRNA interactions. The topology features of proteins and circRNAs are calculated using a novel representation learning approach HIN2Vec across the heterogeneous network. Then, a deep multi-label hierarchical classification model is trained with the topology features to predict the biological process function in the gene ontology for each circRNA. In particular, we manually curated a benchmark dataset containing 185 GO annotations for 62 circRNAs, namely, circRNA2GO-62. The DeepciRGO achieves promising performance on the circRNA2GO-62 dataset with a maximum F-measure of 0.412, a recall score of 0.400, and an accuracy of 0.425, which are significantly better than other state-of-the-art RNA function prediction methods. In addition, we demonstrate the considerable potential of integrating multiple interactions and association networks.

**Conclusions:**

DeepciRGO will be a useful tool for accurately annotating circRNAs. The experimental results show that integrating multi-source data can help to improve the predictive performance of DeepciRGO. Moreover, The model also can combine RNA structure and sequence information to further optimize predictive performance.

## Background

circRNAs are a species of non-coding RNA molecules with closed ring structures, which are highly conserved and unaffected by RNA exonuclease and widely expressed in eukaryotic cells [1–3]. Unlike traditional linear RNAs, circRNA molecules lack 5′-3′ ends and covalently form closed loops, which are not affected by RNA exonuclease, more stable and less prone to degradation [4]. Currently, highly recognized biological functions of circRNA mainly include miRNA sponge, regulatory protein binding, regulatory gene transcription and coding functions, but the main foothold of circRNA research is still miRNA sponge. circRNAs are rich in miRNA binding sites to act as miRNA sponges, preventing miRNA from interacting with mRNA in the 3′ non-translated region, and thus indirectly regulating the expression of downstream target miRNA genes. This mechanism is called competitive endogenous RNA (ceRNA) [5]. Through interacting with biological macromolecules (such as proteins and miRNAs) associated with diseases, circRNA plays an indispensable role in the development of nervous system diseases, musculoskeletal diseases, and cardiovascular diseases. Additionally, circRNA plays a vital regulatory role in the occurrence of cancer, such as participating in the proliferation, migration and invasion of colon, gastric and oesophageal cancer cells [6–9]. Therefore, determining the function of circRNAs in mammalian cells is of great significance for revealing the mechanism of action, diagnosis, and prevention of diseases in physiological and pathological processes.

At present, researchers identify the function of circRNAs through multiple schemes, including pull-down experiments, UV-crosslinked immunoprecipatation (CLIP). Due to the diverse roles and various interacting sites of circRNA, it is challenging to apply biological schemes on a large scale as a result of the time-consuming and costly verification. With the in-depth understanding of circRNA, numerous studies have proven that most circRNAs can flexibly regulate the expression of corresponding genes by interacting with biological macromolecules (such as DNA, proteins, and miRNAs) to achieve their biological functions [10–13]. The existing circRNA function prediction methods, for example, Mireap [14], Miranda [15], TargetScan [16], and FunNet [17], are mainly based on the principle of “guilt-by-association”. These methods elucidate circRNA function by analysing the roles of target genes or promoters. Nevertheless, the predictions of these auxiliary tools are not satisfactory since the majority of the predictions based on the circRNA targets are negative. Some studies [18–20], through GO analysis and KEGG pathway annotation, determined the function of differentially expressed circRNAs between patients and healthy individuals. Through these methods, the functions of circRNAs cannot be identified on a large scale. In recent years, high-throughput sequencing technologies have developed rapidly, and circRNA-related data have grown exponentially. Increasing circRNA co-expression, sequences, interactions and structural information are accumulating. Nevertheless, analyzing and integrating these data remains a challenging task.

In this paper, we present a computational approach, DeepciRGO, for predicting circRNA functions by integrating multiple interactions and associations. DeepciRGO is constructed using the dependencies between GO classes as background information. We first build a global heterogeneous network by integrating circRNA co-expression data, circRNA–protein association data, and protein–protein interaction data. Subsequently, HIN2Vec is utilized to learn embedding vector representations of nodes in the global heterogeneous network. We then feed these features into a novel deep neural notation model, which is constructed to resemble the structure and dependencies between the GO terms, refine the predictions and features at each level of GO, and ultimately optimize the performance of functional predictions based on the performance of the entire ontology hierarchy. In consequence, the maximum F-measure achieves 0.412 on our manually annotated dataset circRNA2GO-62. DeepciRGO outperforms other state-of-the-art function predictive methods in terms of precision, recall and maximum F-measure.

## Results

### Benchmark

At present, there doesn’t exist publicly available circRNA functional annotation database. Therefore, we manually curated functional annotations for circRNAs from the literature. We collected an independent test set of 62 circRNAs (named circRNA2GO-62) containing 185 GO terms (See Additional file 1: Supplementary information for details). Each annotation of circRNA2GO-62 is manually generated and covers most of the available information of circRNAs, including sequences, genomic context, expression, subcellular localization, conservation, functional evidence and other relevant information. Detailed data set can be found in the Additional file 2: Supplementary Table.

### Evaluation measures

In DeepciRGO, the multi-label classifier is used to predict GO terms for a particular circRNA, and each GO term is assigned a probability from 0 to 1. The confidence score indicates the likelihood that the circRNA is annotated with the GO term. The prediction results ultimately depend on the set threshold *k*. Each GO term with the confidence score greater than or equal to *k*, and their ancestors in the GO that have ‘is a’ and ‘has a’ relationships are collected as the set of predictions expressed as *Pc*(*k*) for each threshold *k*. We use *V* to represent the set of GO items that have been experimentally verified. The accuracy of prediction depends on the matching degree of functions predicted and actual functions, which is measured by three widely used statistical indicators: recall, precision and F-measure. In this study, for each circRNA *j* and threshold *k*, precision and recall are defined as follows:

1 $$Prc_{j}(k)= \frac{\sum _{o\in E}T(o\in Pc_{j}(k)\wedge o\in V_{j})}{\sum _{o\in E}T(o\in Pc_{j}(k))},$$

2 $$Rec_{j}(k)= \frac{\sum _{o\in E}T(o\in Pc_{j}(k)\wedge o\in V_{j})}{\sum _{o\in E}T(f\in V_{j})}.$$

In the formula, *o* represents a particular GO term, and *E* represents the whole set of GO terms in the experiment. The definitions of indicator function *T*(*x*) is as follows:

3 $$T(x)= {\left\{ \begin{array}{ll} 1 &{} {x=true} \\ 0 &{} {x=false} \end{array}\right. }.$$

After predicting all circRNAs, we calculate the average accuracy on *h*(*k*) circRNAs, each of which has at least one predicted GO item with the confidence score higher than the threshold *k*. Similarly, the average recall of the whole set of *N* circRNAs can be calculated. The definitions of the average precision and recall are as follows:

4 $$Prc(k)= \frac{1}{h(k)}\cdot \sum _{j=1}^{h(k)}(Prc_{j}(k)),$$

5 $$Rec(k)= \frac{1}{N}\cdot \sum _{j=1}^{N}(Rec_{j}(k)).$$

As for the multi-classification problem, due to the different emphasis of precision and recall, it is difficult to evaluate the model through the two indexes. To solve this problem, we introduce the maximum F-measure, which takes into account both the accuracy and recall of the classifier. The maximum F-measure can be regarded as a harmonic average of precision and recall. Its definition is as follows:

6 $$F_{max}=\max _{k}\left( \frac{2\cdot Prc(k)\cdot Rec(k)}{Prc(k)+Rec(k)}\right) .$$

### Parameter tuning

**Fig. 1:**
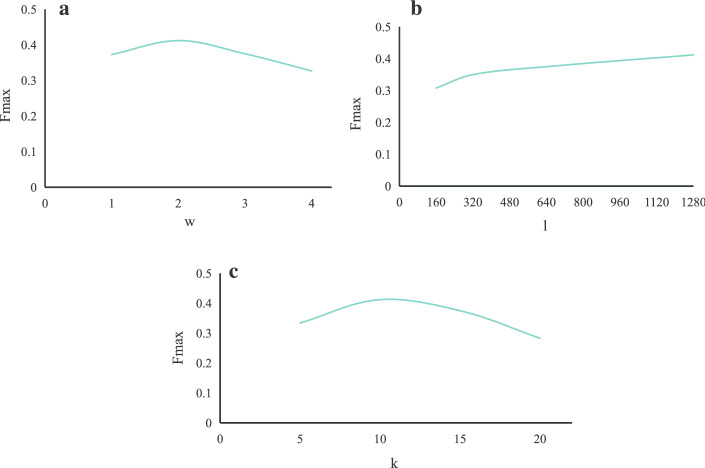
Results with different parameters

Different parameters have an important influence on the predicted results. In HIN2Vec, there are mainly four parameters, namely, the number of steps starting from one node (*k*), the length of the random walks (*l*), the max window length (*w*) and the number of dimensionality (*d*). First, we evaluate *k*, *w*, *l* parameters on the independent test set circRNA2GO-62 by fixing the value of *d*. Figure 1 illustrates the change in $F_{max}$ value when different *k*, *w*, *l* parameters are selected.

The max window length *w*: As shown in Fig. 1a, the performance is first improved and then gradually decreases as *w* increases. And the $F_{max}$ is optimal while *w* is set to 2. Hence, setting *w* to 2 is reasonable.

The length of the random walks *l*: As shown in Fig. 1b, the performance is gradually improved and tends to converge while *l* increases from 160 to 1280. And a longer *l* can generate more data. Thus, to obtain more data, we set *l* to 1280.

The number of steps starting from one node *k*: As shown in Fig. 1c, the performance is significantly improved when *k* is increased to 10. However, it gradually decreases when *k* is set from 15 to 20. Therefore, we set *k* to 15 in this work.

Based on the above results, we set the three parameters *l*, *w*, and *k* to 1280, 2, and 15, respectively.

**Fig. 2:**
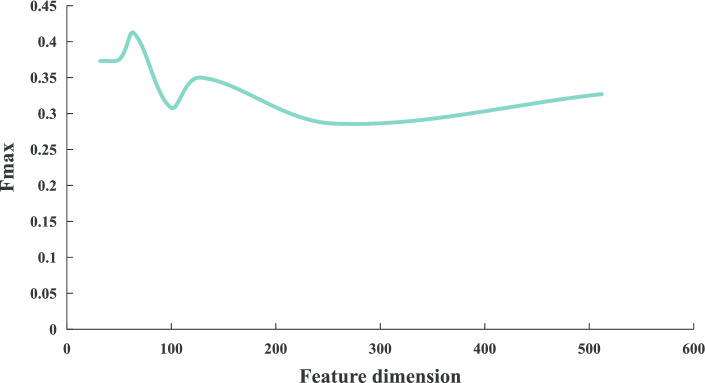
The Fmax values when using different dimensions

Finally, in order to determine the optimal dimension, we pre-assign the other three parameters ($k = 10$, $l = 1280$, $w = 2$) and then continuously change its value on the benchmark dataset circRNA2GO-62 to evaluate the predictive performance. Figure 2 illustrates the change of the $F_{max}$ values when the node feature dimensions range from 32 to 512. Experimental results demonstrate that the overall performance of $F_{max}$ reaches the highest when the dimensions of the feature vector is set to 64. Hence, 64-dimensional feature vectors are selected to construct the DeepciRGO classification model.

### The impact of integrating multi-source data

**Fig. 3:**
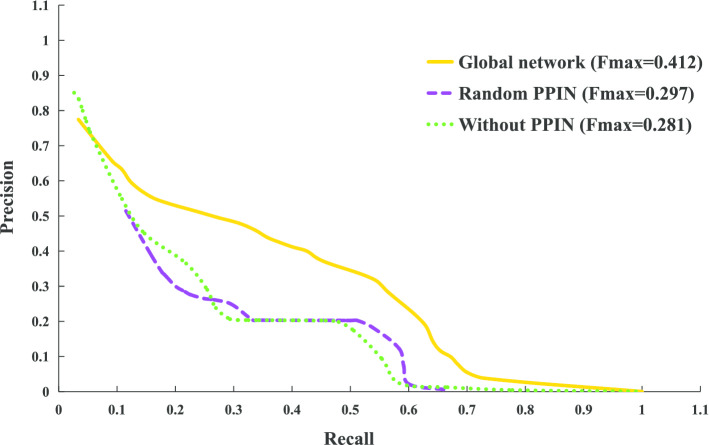
The precision–recall curves of circRNA2GO-62 biological process prediction on different networks (global network, without PPI network and random PPI network)

In our method, the integration of protein interactions contributes to the functional annotations of circRNAs. To verify this, we compare the performance on two different network configurations, namely, the global network (including PPI) and the PPI-free network (all PPIs are removed). The results are shown in Fig. 3 when the parameters (*k*, *l*, *w*, *d*) are set to 10, 1,280, 2 and 64, respectively. The global heterogeneous network constructed by integrating multiple data sources (including of circRNA co-expression data, circRNA–protein interaction data, and PPI data) is better than the PPI-free network, with the $F_{max}$ of 0.412 and 0.281 respectively. The performance is improved by approximately 47% as PPI data is integrated. This experiment demonstrates that integrating multiple interaction and association networks can significantly improve the performance of predicting circRNA function.

To further evaluate the impact of multi-source data on performance, we randomly generate the same number of associated entries between the protein pairs as the original PPI data (The global network and the random PPI network can be found in Additional file 1: Supplementary information). Experiments confirm that the performance of the stochastic integrated network we built later is significantly reduced (See Table 1 for details). Its $F_{\max }$ is 0.297, which is lower than that of the global network but higher than that of the PPI-free network. These results prove the benefit for integration of multi-source data.

**Table 1:** Performance comparison of circRNA2GO-62 biological process prediction on different networks (global network, without PPI network and random PPI network)

Method	Precision	Recall	Fmax
Global network	0.754	0.689	0.412
Random PPIN	0.559	0.202	0.297
Without PPIN	0.458	0.202	0.281

### Comparison of graph embedding methods

Graph embedding is the method of representing nodes in a network with low-dimensional, dense, real-valued vectors. The core idea is to project heterogeneous information into the same low-dimensional space to facilitate downstream computation, such as tag recommendation [21], vertex classification [22, 23] and link prediction [24, 25]. Recently, a number of methods have been developed to extract the latent representations of networks. In this work, we choose four different network representation algorithms for comparison: DeepWalk [26], HIN2Vec [27], Struc2vec [28] and Metapath2vec [29]. To be fair, we use the same global network, multi-classification model and benchmark built above. As shown in Table 2, HIN2Vec is significantly superior to DeepWalk, metapath2vec and struc2vec in BP terms of $F_{\max }$. Therefore, HIN2Vec is selected to learn the low-dimensional potential representation of nodes in the heterogeneous network.

**Table 2:** Performance comparison of different network representation algorithms in different dimensions

Method	Dimension			
	64	128	256	512
DeepWalk	0.352	0.329	0.278	0.228
Struc2vec	0.275	0.291	0.297	0.271
Metapath2vec	0.195	0.251	0.111	0.214
Hin2Vec	0.412	0.350	0.286	0.283

### Performances

To better assess the performance of DeepciRGO, we compare it with three existing models: KATZLGO [30], PmiRGO [31] and BiRWLGO [32]. KATZLGO and BiRWLGO are two link-based prediction methods that infer the functions of RNAs by calculating the correlation scores between lncRNAs and proteins in a global network. PmiRGO is a machine learning method that predicts the functions of miRNAs by training a classifier according to the topological features and GO annotations of proteins. We implement the three methods on the circRNA2GO-62 dataset and perform comparison with DeepciRGO. The performance is only evaluated in terms of biological process (BP) since most annotations in circRNA2GO-62 are BP terms.

**Fig. 4:**
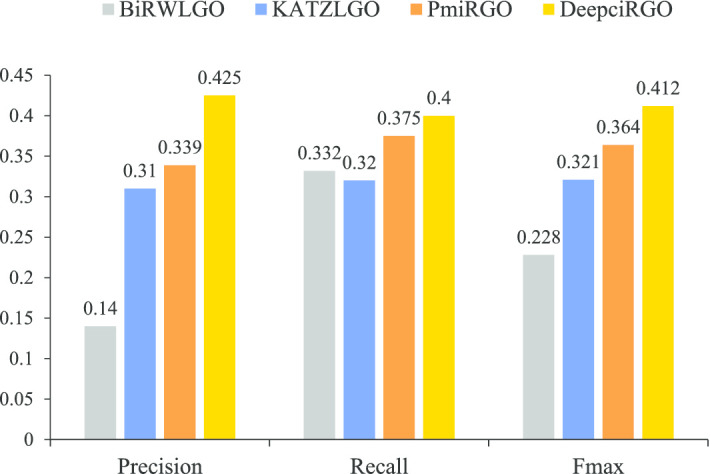
Comparison of the performance of DeepciRGO and other existing methods in BP terms of recall, precision and $F_{max}$

**Fig. 5:**
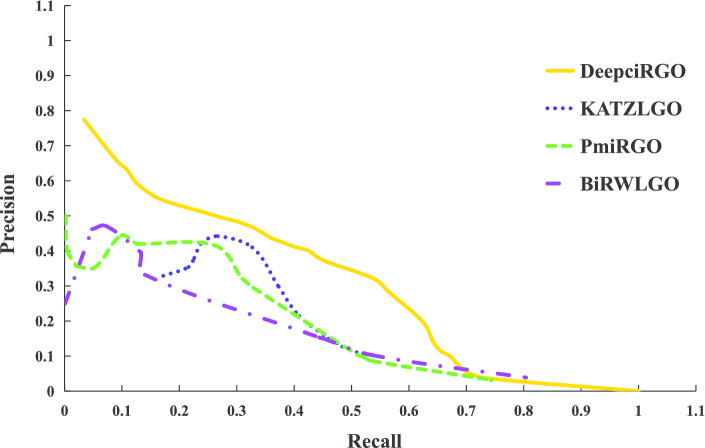
Precision–recall curves of DeepciRGO and other methods (BiRWLGO, PmiRGO and KATZLGO) on the circRNA2GO-62 dataset for BP terms

**Fig. 6:**
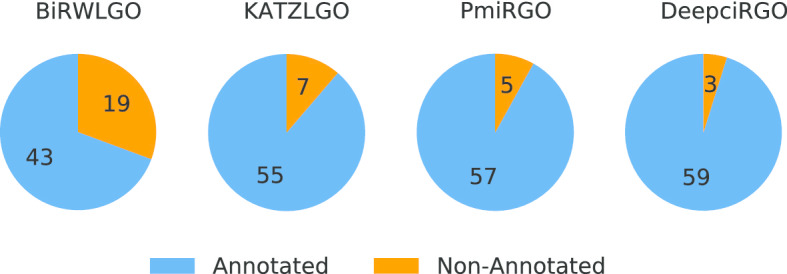
Performance comparison of coverage on the independent dataset circRNA2GO-62 by DeepciRGO and the other three methods (PmiRGO, KATZLGO and BiRWLGO)

The results of different methods on circRNA2GO-62 are illustrated in Fig. 4. The performance of DeepciRGO is significantly better than the other three methods. DeepciRGO achieves the best $F_{max}$ score of 0.412. For recall and precision, our method also reaches 0.400 and 0.425, respectively. Figure 5 shows the precision–recall curves for these four methods on the circRNA2GO-62 dataset. As we can see, the curve of DeepciRGO is almost above the curves of other methods. When recall is less than 0.68, the performance of DeepciRGO is significantly better than that of the other three methods. The DeepciRGO still achieves comparable performance with other methods while recall $> 0.68$. We also compare these models by calculating the number of circRNAs correctly predicted on BP terms. As shown in Fig. 6, DeepciRGO successfully annotates 59 circRNAs from the circRNA2GO-62 dataset, again significantly higher than 57 of PmiRGO, 55 of KATZLGO, and 43 of BiRWLGO. All the results demonstrate that DeepciRGO, using HIN2Vec to extract the topology of the global network, can greatly improve the prediction performance of circRNA function.

### Case study: SHPRH

Hsa_circ_0001649 is produced at the SHPRH gene locus containing exon 26–29 [33]. Research indicates that hsa_circ_0001649 is significantly down regulated in hepatocellular carcinoma (HCC) and may function in tumorigenesis and metastasis of HCC. Xu et al. [34] explored the role of circRNA hsa_circ_0001649 in the regulation of proliferation, migration and invasion of cholangiocarcinoma cells. Wound healing and transwell assays showed that inhibition of hsa_circ_0001649 significantly improved the migration and invasion ability of human bile duct cancer cells. Flow cytometry analysis and AO/EB double fluorescence staining assays suggested that the proliferation effects of hsa_circ_0001649 on colon cancer cell-associated antigens is partly due to changes in apoptosis. Hsa_circ_0001649 is also involved in the regulation of matrix metalloproteinase expression.

In our study, we annotated a total of 79 GO terms of biological process for hsa_circ_0001649 through DeepciRGO. The top 15 GO terms of the SHPRH gene are shown in Table 3. For biological processes, most terms are involved in the regulation of cell proliferation, migration, and apoptosis, as well as the regulation of gene expression, such as GO:0008285 (negative regulation of cell population proliferation), GO:0030336 (negative regulation of cell migration), GO:0051050 (positive regulation of transport), GO:2000147 (positive regulation of cell motility), GO:0040017 (positive regulation of locomotion), GO:0045597 (positive regulation of cell differentiation), GO:0010647 (positive regulation of cell communication), GO:0051272 (positive regulation of cellular component movement) and GO:0010468 (regulation of gene expression). These results prove that our method can predict the function of SHPRH relatively successfully.

**Table 3:** The top 15 predicted BP functions for circRNA SHPRH by DeepciRGO

Rank	GO term	GO name
1	GO:0010468	Regulation of gene expression
2	GO:0060255	Regulation of macromolecule metabolic process
3	GO:0019222	Regulation of metabolic process
4	GO:0023056	Positive regulation of signalling
5	GO:0023051	Regulation of signalling
6	GO:0048585	Negative regulation of response to stimulus
7	GO:0010647	Positive regulation of cell communication
8	GO:0051272	Positive regulation of cellular component movement
9	GO:0045597	Positive regulation of cell differentiation
10	GO:0048583	Regulation of response to stimulus
11	GO:0008285	Negative regulation of cell population proliferation
12	GO:0030336	Negative regulation of cell migration
13	GO:0051050	Positive regulation of transport
14	GO:2000147	Positive regulation of cell motility
15	GO:0040017	Positive regulation of locomotion

## Discussion

Currently, although thousands of circular RNAs have been identified from different cell types in several model organisms using RNA-seq technology, the biological functions of most circular RNAs remain unknown. In addition to biological experiments, computational methods provide another method for researching the function of circRNAs. However, designing accurate, reliable, and efficient circRNA function annotation methods are still a challenge, far from reaching the actual level of large-scale applications, and there are still many technical difficulties to be overcome. Base on this, we build a new circRNA function prediction model by combining network characteristics and deep learning. Due to the lack of a standard database of known human circRNA annotations, we downloaded protein annotations from Uniprot-Goa 201010 as a training set. Then, the trained DeepciRGO model was evaluated using the artificial aided circRNA2GO-62. At the same time, DeepciRGO still has better performance compared with other advanced methods.

The main novelty of this approach lies in the following three points: First, there are no circRNA function annotation datasets for training an ML model. Here, we build the training dataset by constructing a heterogeneous network and extracting the representations of nodes. This opens up a new avenue to predict the functions of circRNAs. Second, we manually annotate the circRNA to build our test set (named circRNA2GO-62) by reviewing and collecting some articles on current research circRNA and providing corresponding functions. Third, we build a new circRNA function prediction model by combining network characteristics and deep learning.

## Conclusions

In this study, we propose a computational approach, DeepciRGO, to predict the function of circRNA by integrating multiple circRNA-related biological information. First, we construct a global heterogeneous network according to circRNA co-expressions, circRNA–protein associations, and protein–protein interactions. Then, the latent topological features of the global network are extracted through HIN2Vec and are further fed into a deep neural network classifier. Finally, circRNAs are annotated with GO terms through the trained classifier. In terms of performance, we perform independent tests on the manually processed standard dataset. The results demonstrate that DeepciRGO outperforms other advanced methods in terms of precision, recall and $F_{max}$. In addition, the PPI data can help to improve the predictive performance for circular RNAs.

We believe that DeepciRGO can combine sequence, disease association and structural information to more accurately predict the functions of circRNAs, which is also an excellent tool for revealing the mechanism of circRNAs in both physiological and pathological processes. At the same time, we will continue to add miRNA-circRNA interaction data in the follow-up work to improve our model and apply it to the functional annotation of circRNA of other species to make it have a better generalization ability. Additionally, this model can be used to predict disease association of genes encoded by the disease ontology, or phenotypic association of genetic variations encoded by the phenotypic ontology [35].

## Methods

### Datasets and pre-processing

#### circRNA co-expression similarity

The establishment of circRNA similarity network is based on the basic biological hypothesis that genes within the group have similar expression profiles, which may have similar functions. circRNA-circRNA related data is relatively scarce because there is no standard database available. The CircRiC and MiOncoCirc databases [36, 37] also only contain data on circRNA expression characteristics, biological formation, drug response, and integrated analysis, without the circRNA expression profile information we need. Therefore, we collect relevant data by reviewing the research-proven literature. We finally obtain circRNA expression profiling data from Peng et al.’s work [38], which consists of expression profiles of 2932 circRNAs. In the field of natural science, Pearson correlation coefficient is widely used to measure the degree of correlation between two variables and can well reflect the relationship between them, with its value between -1 and 1. So, the Pearson correlation coefficient (PCC) between each pair of circRNAs is calculated based on the downloaded expression profile information and used to construct the circRNA similarity network.

#### circRNA–protein associations

The circRNA–protein data is downloaded and compiled from the StarBase v2.0 database and CSCD http://gb.whu.edu.cn/CSCD/ [39, 40]. The two databases integrate almost all published circRNA related data, which contains the circRNA–protein interactions derived from biological experiments, text mining, and computational prediction methods. To ensure the reliability of data, we remove the duplicate entries from the circRNA–protein associations and delete entries that don’t exist in the protein–protein associations and circRNA-circRNA associations according to the protein ID and circRNA ID. Finally, a total of 2,932 circRNAs and 18,348 target genes with 188,479 circRNA-target interactions among them are screened, which are used to construct the circRNA–protein interaction network.

#### Protein–protein interactions

At present, there are many databases of protein interactions. But the STRING V10.0 [41] database is the one that covers the most species and interaction information. The interactions in the database are derived not only from biological experiments, but also from text mining and algorithm models. After removing the duplicate entries from the protein–protein interactions and deleting entries that don’t exist in the circRNA–protein associations according to the protein ID, we obtain a total of 5,172,245 interactions containing 18,348 proteins. Each pair of interaction has a confidence score which is computed by combining the probabilistic integrals of single-channel array and double-channel array.

**Fig. 7:**
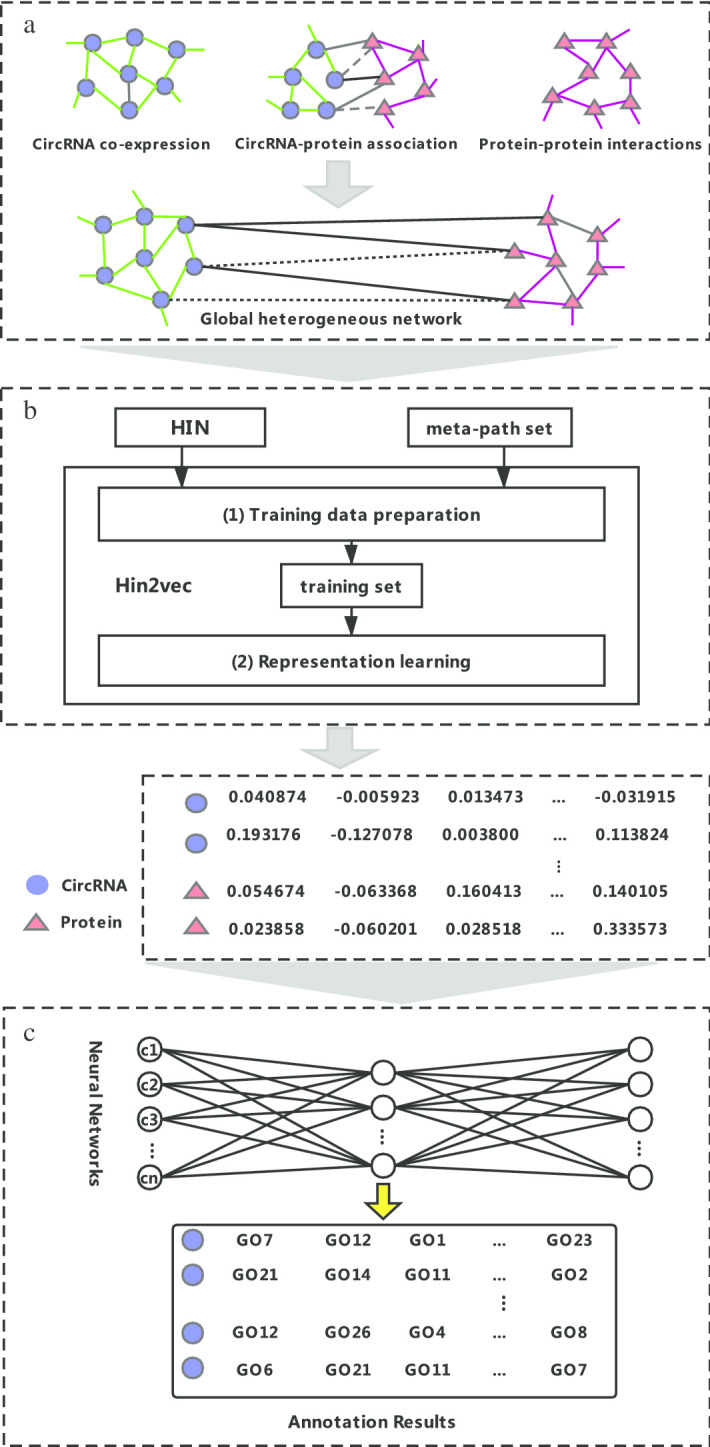
Flowchart of DeepciRGO, which includes three steps: (**a**) build the global heterogeneous network by integrating three networks (circRNA co-expression network, circRNA–protein interaction network, and PPI network); (**b**) employ HIN2Vec to learn the latent representations of the nodes (circRNAs and proteins); (**c**) train each GO class with the neural network model and annotate circRNAs

The overall flow of our method is shown in Fig. 7. It includes three steps. (a) Construct the global heterogeneous network according to the circRNA co-expression similarity, circRNA–protein associations, and PPIs. (b) Employ the HIN2Vec algorithm [27] to extract the representation of each node in the global heterogeneous network. (c) Train the multi-label neural network and apply it to our manually curated independent test dataset.

### Build the global heterogeneous network

In this work, we collect circRNA expressions, circRNA–protein associations, and protein–protein interactions from different databases. In total, 2932 circRNAs and 18,348 protein-coding genes are screened. Based on the data, we build a global heterogeneous network to represent the biological entities and the relationships among nodes since heterogeneous networks usually contain very rich information. In addition, there is evidence that circRNAs most likely have the same or similar functions with the associated proteins [10]. Therefore, we construct a global network to annotate the functions of circRNAs.

### Obtain vector representations of nodes

In order to capture the rich semantics of embedding in a heterogeneous networks, it is necessary to use appropriate network representation learning methods to extract embedded information in the network structure, while preserving the original relationships among nodes as input features of deep neural network models. In this study, HIN2Vec is used to learn low-dimensional vector representations of circRNAs and proteins in the heterogeneous network [27]. The core of HIN2Vec is a neural network model, which can learn not only the representations of nodes in the network, but also the representations of relationships (meta-paths).

The basic idea of the HIN2Vec model is to target multiple prediction tasks, each task corresponding to a meta-path, jointly learning a neural network model, predicting a set of target relationships between any given pair of nodes, thereby learning the low-dimensional vector representation of each node. As shown in step B in Fig. 7, HIN2Vec model is specifically divided into two stages: (1) training data generation and (2) representation learning. In the data generation part, random walk and negative sampling are employed to extract the training data in the form of (*a*, *b*, *B*(*a*, *b*, *z*)) through sampling the HIN. Here, *a* and *b* represent two nodes, *z* is the relationship between the two nodes, and *B*(*a*, *b*, *z*) is a binary value indicating whether there is a relationship *z* between *a* and *b*.

**Fig. 8:**
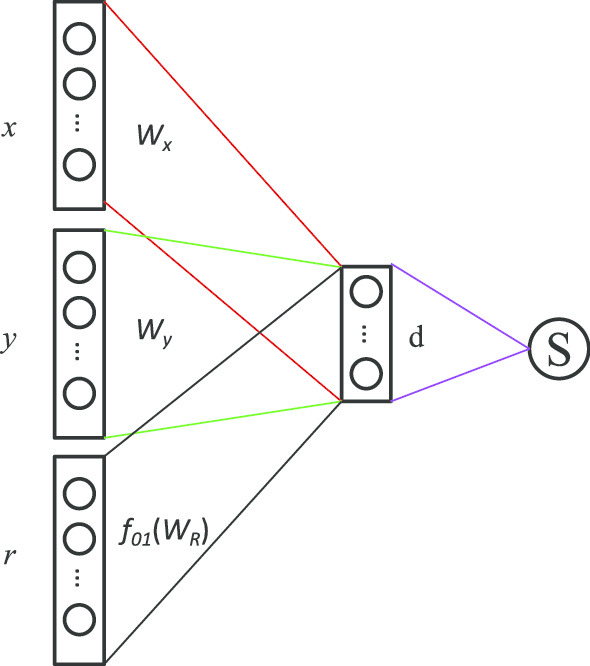
The HIN2Vec NN model

In the second step, the representations of nodes are learned by building a binary classifier to predict whether there is a definite relationship *z* between two nodes *a* and *b*. In HIN2Vec, a three-layer feedforward neural network (NN) model serves as the binary classifier (as shown in Fig. 8). The HIN2Vec model takes nodes *a*, *b* and their specific relationship *z* as input to predict whether the relationship *z* exists between them. The input layer of the model is fed by three one-hot encoded vectors, $\vec {a}$, $\vec {b}$ and $\vec {z}$, denoting *a*, *b*, and *z*, respectively. Then, in the latent layer, they are converted to the hidden vectors $W^{'}_{A}\vec {a}$, $W^{'}_{B}\vec {b}$ and $f_{01}(W^{'}_{Z}\vec {z})$. Since the semantic meaning of relation and node is different in the learning process, we regularize the relation vector *z* with the regularization function $f_{01}(.)$ to enhance its generalization ability, which limits the value of relation vector *z* between 0 and 1. The three vectors are aggregated, and denoted by $W^{'}_{A}\vec {a}\odot W^{'}_{B}\vec {b}\odot f_{01}(W^{'}_{Z}\vec {z})$ through the Hadamard function and identity function for activation. In the output layer, the HIN2Vec NN model takes the summation function and the sigmoid function, namely, sigmoid $\left( \sum W'_A\overrightarrow{a}\odot W'_B\overrightarrow{b}\odot f_{01}\left( W'_Z\overrightarrow{z}\right) \right)$, as the input function and activation function, respectively.

HIN2Vec is iteratively trained on the training set *T* through the back-propagation algorithm based on random gradient descent. The weights in $W_{A}$, $W_{B}$, and $W_{Z}$ for each entry in *T* are adjusted constantly by maximizing the objective function *F*, which is the multiplication of $F_{a,b,z}(a,b,z)$ for each training data entry in *T*. The objective function *F* and derivation of *logF* are defined as:

7 $$\begin{aligned} F\propto logF=\Sigma _{a,b,z\in T}logF_{a,b,z}(a,b,z). \end{aligned}$$

Specifically, for a training data entry $\langle a,b,z, R(a,b,z)\rangle$, when *R*(*a*, *b*, *z*) is 1, $F_{a,b,z}(a,b,z)$ aims to maximize *P*(*z*|*a*, *b*); otherwise $F_{a,b,z}(a,b,z)$ aims to minimize *P*(*z*|*a*, *b*). Thus, $F_{a,b,z}(a,b,z)$, *P*(*z*|*a*, *b*) and $logF_{a,b,z}(a,b,z)$ are written as follows:

8 $$\begin{aligned}&F_{a,b,z}(a,b,z)= {\left\{ \begin{array}{ll} P(z|a,b), &{} {if \,R(a,b,z)=1} \\ 1-P(z|a,b), &{} {if\, R(a,b,z)=0} \end{array}\right. }, \end{aligned}$$

9 $$\begin{aligned}&logF_{a,b,z}(a,b,z)=R(a,b,z)logP(z|a,b) \\&\quad +[1-R(a,b,z)]log[1-P(z|a,b)], \end{aligned}$$

10 $$\begin{aligned}&P(z|a,b)=sigmoid(\sum (W^{'}_{A}\vec {a}\odot W^{'}_{B}\vec {b}\odot f_{01}(W^{'}_{Z}\vec {z}))). \end{aligned}$$

After training, the representations of nodes in the global heterogeneous network are extracted. The result is an N $\times$ M matrix, where *N* represents the total number of circRNAs and proteins in the network, and each biological entity is represented by a *M*-dimensional vector.

### Training multi-classification model

**Fig. 9:**
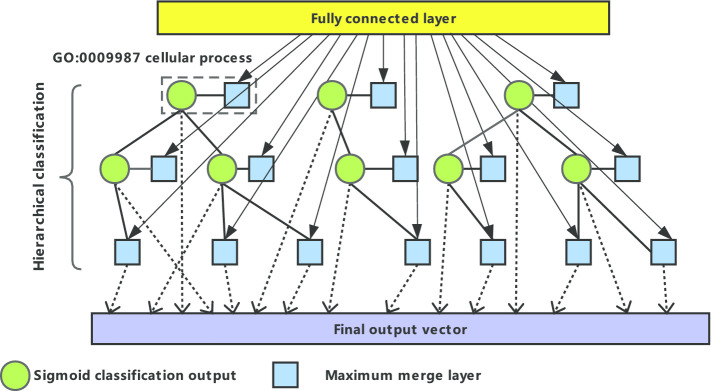
the hierarchical architecture of classification in the neural network model

Gene ontology (GO) contains three functional information of gene involved in biological process, cell location and molecular function, which organizes different functional concepts into directed acyclic graph (DAG) structure. The GO graph has the nature of a classification tree. Different from tree, the nodes in the GO graph may not only have multiple child nodes, but may also have multiple parent nodes, and have different relationships with different parent nodes. Therefore, predicting the GO terms of circRNA can be considered as a hierarchical multi-label classification problem [42]. In DeepciRGO, we establish a multi-label classification model combining neural network and symbol intelligence for each class in GO terms, which deeply integrates the respective advantages of neural system and symbol system (Fig. 9). This hierarchical classification model takes the topological characteristics of circRNAs and protein in heterogeneous networks as input and is trained step by step. We create a binary marker vector for each training sample. If the training sample has a GO annotation in our selected class list, we mark the position of the corresponding item in the binary marker vector as 1; otherwise, it is marked as 0. Each neural network of DeepciRGO contains a fully connected layer and a sigmoid activation function. The output vectors of the previous fully connected layer are fed into the next layer. It is important to note that all neural networks share the low-dimensional features of the first full connection layer. To ensure the consistency of GO items hierarchy classification, we build a maximum merge layer for each GO item with child nodes in the model. The merge layer selects the value with the highest predicted score among GO items and all their sub items. In consequence, the final output vector of the hierarchical multi-label classification model is the series of activations of the leaf nodes and the maximum merge layers of the non-leaf nodes.

Due to the lack of a standard database of known human circRNA annotations, we download protein annotations from UniProt-GOA version 201010 as the training set. In training the DeepciRGO, we perform 5-fold cross-validation and use multi-class cross entropy to calculate the loss function [43]. Then, we employ the RMSProp optimizer to optimize the model. The initial weight of the model is initialized based on a uniform distribution. At the end of each training epoch, the weight of the entire network is automatically adjusted by backpropagation. To accelerate the training process, we use NVIDIA Pascal X GPUs. The training time for the model is less than two hours and the inference time is less than one second. To prevent overfitting of the model, we use a dropout layer as the regularizer. We manually adjust the following parameters: batch size, number of connected neurons, and learning rate. We select the optimal parameters depending on the values of validation loss. Table 4 shows the validation losses and train losses for different embedding sizes. Through the continuous adjustment, we finally obtain the optimal model with a minimum batch size of 64 and learning rate of 0.01.

**Table 4:** Validation losses and train losses of the models for different embedding sizes

Embedding size	Val_loss	Loss
32	0.0754	0.0689
64	0.0751	0.0664
128	0.0757	0.0665
256	0.0767	0.0674

In summary, we build a machine learning framework to predict the function of circRNAs. The first part of the framework extracts the topological information of each node in the global network as its feature. The second part builds a neural network for each GO, considering the functional dependencies between the classes in GO. The purpose is that the framework can identify both explicit dependencies between classes in GO and implicit dependencies (such as frequently co-occurring classes).

## Supplementary information

Additional file 1. The benchmark dataset containing185 GO annotations for 62 circRNAs(circRNA2GO-62),which wheremanually curated according to the information including genomiccontext, sequences, expression, structural information,conservation, subcellular localization, functional evidence.

Additional file 2. The biological process (BP)functions and case studies of circRNAs predicted by DeepciRGO.

## Data Availability

The source code and data sets of DeepciRGO are freely available at http://denglab.org/DeepciRGO/.

## Abbreviations

CLIP: UV-crosslinked immunoprecipatation
BP: Biological process
KEGG: Kyoto Encyclopedia of Genes and Genomes
GO: Gene ontology
PPI: Protein–protein interaction
DAG: Directed acyclic graph
NN: Neural network
HIN: Heterogeneous information networks
PCC: Pearson’s correlation coefficient
PR: Precision–recall
HCC: hepatocellular carcinoma
AO/EB: Acridine orange/ethidium bromide
