# Supplementary material for: DeepciRGO: functional prediction of circular RNAs through hierarchical deep neural networks using heterogeneous network features
Source: BMC Bioinformatics. 2020 Nov 12;21:519. doi: 10.1186/s12859-020-03748-3 (PMC7659092; doi:10.1186/s12859-020-03748-3)
Supplement: Supplementary file 1 — The benchmark dataset containing185 GO annotations for 62 circRNAs(circRNA2GO-62),which wheremanually curated according to the information including genomiccontext, sequences, expression, structural information,conservation, subcellular localization, functional evidence. [file 12859_2020_3748_MOESM1_ESM.pdf]

SUPPLEMENTARY MATERIALS

DeepciRGO: Functional prediction of circular RNAs based on the heterogeneous networks

Lei Deng , Wei Lin , Jiacheng Wang and Jingpu Zhang

1 Experimental result

When we manually annotated independent test data sets (circRNA2GO-62), we found that circRNA's functions in cells were mostly biological processes (BP), and only a small part were cellular components (CC) and molecular functions (MF). So, in order to better evaluate our model, we only did comparative experiments on biological processes. Given a target circRNA, the candidate GO terms are predicted and ranked by DeepciRGO. In order to compare whether the predicted GO terms are consistent with the circRNA function confirmed by experiments, we sorted the predicted GO according to the predicted score and evaluated the top 20 GO terms of BP.

1.1 Case study:CircMYLK

CircMYLK, also known as hsa_circ_0002768, is spliced from the MYLK gene [1]. By screening circRNA expression profiles, Zhong et al. found that the circMYLK levels were significantly increased in bladder cancer tissue samples than in matched normal bladder tissue samples and that the circMYLK levels were correlated with bladder cancer stage [2]. In bladder cancer cells studied in vitro, upregulation of circMYLK enhanced tumor cell growth, migration and apoptotic process[3], whereas circMYLK knockdown resulted in the opposite effects. In vivo studies showed that circMYLK upregulation promoted the growth of xenograft tumors [2]. Further studies have shown that circMYLK regulates the expression of vascular endothelial growth factor A (VEGFA), induces epithelial–mesenchymal transition (EMT), and activates Ras/ERK signaling [2].

CircMYLK are annotated with 64 GO terms of biological process in total through DeepciRGO. The top 20 GO terms assigned to the CircMYLK are shown in Table S1. For biological process, most terms are involved in the regulation of cell migration, cell growth, vasculature development as well as regulation of gene expression. such as GO:0030336 (negative regulation of cell migration), GO:0030307 (positive regulation of cell growth), GO:0040008 (regulation of growth), GO:1901342 (regulation of vasculature development). The predicted function of CircMYLK by DeepciRGO is basically consistent with the experimental results supported by the references.

Table S1. The top 20 predicted BP functions for CircMYLK (namely hsa_circ_0002768), which annotated with 64 GO terms of biological process in total by DeepciRGO.

rank	GO term	GO name

1	GO:1902589	organelle organization
2	GO:0043065	positive regulation of apoptotic process
3	GO:0016043	cellular component organization
4	GO:0071840	Cellular component organization or biogenesis
5	GO:0010468	regulation of gene expression
6	GO:0010556	regulation of macromolecule biosynthetic process
7	GO:0008285	negative regulation of cell population proliferation
8	GO:0009966	regulation of signal transduction
9	GO:0030336	negative regulation of cell migration
10	GO:0032268	regulation of cellular protein metabolic process
11	GO:0051246	regulation of protein metabolic process
12	GO:0032268	regulation of cellular protein metabolic process
13	GO:0031326	regulation of cellular biosynthetic process
14	GO:1901342	regulation of vasculature development
15	GO:0035821	modification of morphology or physiology of other organism
16	GO:0051047	positive regulation of secretion
17	GO:1903532	positive regulation of secretion by cell
18	GO:0030307	positive regulation of cell growth
19	GO:0045927	positive regulation of growth
20	GO:0040008	regulation of growth

1.2 Case study:circZNF609

Studies have shown that circZNF609 could be translated into a protein, which was involved in controlling myoblast proliferation.[4] Meanwhile,circZNF609 increased oncogene p70S6K1 expression by sponging and sequestering miR-145-5p activity. Moreover, ectopic expression of miR-145-5p or silencing of p70S6K1 could effectively reverse circZNF609-induced promotion of breast cancer proliferation, migration, and invasion.[5] cZNF609 silencing decreased retinal vessel loss and suppressed pathological angiogenesis in vivo. cZNF609 silencing increased endothelial cell migration and tube formation, and protected endothelial cell against oxidative stress and hypoxia stress in vitro.[6]

In our study, circZNF609 are annotated with 48 GO terms of biological process in total through DeepciRGO. The top 20 GO terms assigned to the circZNF609 are shown in Table S2. For biological process, most terms are involved in the regulation of cell migration, cell growth and vasculature development. such as GO:0030335 (positive regulation of cell migration), GO:0030307 (positive regulation of cell growth), GO:0045927 (positive regulation of growth), GO:0040008 (regulation of growth), GO:1901342 (regulation of vasculature development). These data also show that the predicted circRNA functions of our model are basically consistent with those confirmed by experimental studies.

Table S2. The top 20 predicted BP functions for circZNF609 (namely hsa_circ_0000615), which annotated with 48 GO terms of biological process in total by DeepciRGO.

Rank	GO term	GO name
1	GO:0010628	positive regulation of gene expression
2	GO:0010604	positive regulation of macromolecule metabolic process
3	GO:0009893	positive regulation of metabolic process
4	GO:0071840	Cellular component organization or biogenesis
5	GO:0010468	regulation of gene expression
6	GO:0030335	positive regulation of cell migration
7	GO:2000147	positive regulation of cell motility
8	GO:0040017	positive regulation of locomotion
9	GO:0030336	negative regulation of cell migration
10	GO:0051272	positive regulation of cellular component movement
11	GO:0030307	positive regulation of cell growth
12	GO:0045927	positive regulation of growth
13	GO:0040008	regulation of growth
14	GO:0051093	negative regulation of developmental process
15	GO:0035821	modification of morphology or physiology of other organism
16	GO:1901342	regulation of vasculature development
17	GO:0051128	regulation of cellular component organization

18	GO:0050793	regulation of developmental process
19	GO:1903532	positive regulation of secretion by cell
20	GO:0051239	regulation of multicellular organismal process

References

[1] Glažar P, Papavasileiou P, Rajewsky N. circBase: A database for circular RNAs. RNA. 2014;20(11):1666–70.

[2] Zhong Z, Huang M, Lv M, et al. Circular RNA MYLK as a competing endogenous RNA promotes bladder cancer progression through modulating VEGFA/VEGFR2 signaling pathway. Cancer Lett. 2017;403:305–17.

[3] Zhong Z, Huang M, Lv M, et al. Circular RNA MYLK as a competing endogenous RNA promotes bladder cancer progression through modulating VEGFA/VEGFR2 signaling pathway[J]. Cancer letters, 2017, 403: 305-317.

[4] Legnini I, di Timoteo G, Rossi F, et al. Circ-ZNF609 is a circular RNA that can be translated and functions in myogenesis. Mol Cell. 2017;66(1):22 – 37.

[5] Wang S, Xue X, Wang R, et al. CircZNF609 promotes breast cancer cell growth, migration, and invasion by elevating p70S6K1 via sponging miR-145-5p[J]. Cancer management and research, 2018, 10: 3881.

[6] Liu C, Yao M D, Li C P, et al. Silencing of circular RNA-ZNF609 ameliorates vascular endothelial dysfunction[J]. Theranostics, 2017, 7(11): 2863.

2 Dataset

Since there is no available public database of lncRNA function annotations, we manually curate a independent test set of 62 circRNAs with 185 GO terms (named as circRNA2GO-62) based on references. Each annotation of circRNA2GO-62 is manually curated and contains referenced information about the circRNA, including sequences, genomic context, expression, subcellular localization, conservation, functional evidence and other relevant information.

Table S3. The independent test set of 62 circRNAs with 185 GO terms (named as circRNA2GO-62) based on references.

circRNA id	pmid	GO terms
hsa_circ_0000069	28003761	GO:0008284,GO:0070317,GO:0030335
hsa_circ_0000096	28089832	GO:0035198,GO:0030336
hsa_circ_0000140	25689795	GO:1903354
hsa_circ_0000181	28940688	GO:1903356,GO:2000401,GO:0045595
hsa_circ_0000284	28794202	GO:2000402,GO:0045766,GO:0035198,GO:0030335,GO:0045766
hsa_circ_0000284	27050392	GO:0008284,GO:0030308
hsa_circ_0000523	30403259	GO:0043066,GO:0030177,GO:0035198
hsa_circ_0000523	25624062	GO:0008285
hsa_circ_0000594	28219405	GO:0008284,GO:0030335,GO:0043066
hsa_circ_0000615	30288120	GO:0035198,GO:0010628,GO:0030335,GO:0030307
hsa_circ_0000615	28824721	GO:0090051,GO:1900408
hsa_circ_0000677	29928432	GO:0030177,GO:0035198
hsa_circ_0000677	27058418	GO:0008284
hsa_circ_0001313	28249903	GO:0042127,GO:0030334
hsa_circ_0001445	28765045	GO:0030308,GO:0030336,GO:0035198,GO:0030177,GO:0006281,GO:0006260,GO:0045787,GO:0043066
hsa_circ_0001649	29337065	GO:0030336,GO:0043065
hsa_circ_0001649	26600397	GO:0008285
hsa_circ_0001724	29207676	GO:0008285,GO:0030336,GO:0035198
hsa_circ_0001727	28211215	GO:2000346,GO:0030336
hsa_circ_0001821	29693417	GO:0010628,GO:0043066,GO:0030307
hsa_circ_0001821	27986464	GO:0013598,GO:0008284
hsa_circ_0001824	28484086	GO:0035198
hsa_circ_0001875	28484086	GO:0035198,GO:0014068

hsa_circ_0001946	28174233	GO:0014067,GO:0051898,GO:0008285,GO:0030336,GO:0030307,GO:1901224,GO:0045669,GO:0032024,GO:0035821,GO:0035198,GO:0010468
hsa_circ_0002062	28682884	GO:0035198,GO:1901186
hsa_circ_0002138	25624062	GO:0008285
hsa_circ_0002768	28687357	GO:0070374,GO:0030949,GO:0010718,GO:1903589,GO:0010468,GO:0035198,GO:0043065,GO:0008284,GO:0030335,GO:1900748
hsa_circ_0003159	28618205	GO:1903356
hsa_circ_0003221	29125888	GO:0008284,GO:0030335
hsa_circ_0003222	30520539	GO:2000774,GO:1901798
hsa_circ_0003222	28893265	GO:0035198， GO:0008285
hsa_circ_0004018	28938566	GO:0045595,GO:0035198
hsa_circ_0004214	28622299	GO:0046830,GO:0008284,GO:0010718,GO:0030335
hsa_circ_0004383	27613831	GO:0060253 GO:0045786,GO:0030111
hsa_circ_0004619	28484086	GO:0035198,GO:0043065
hsa_circ_0004712	28983619	GO:0035198,GO:0010468
hsa_circ_0004771	28484086	GO:0035198
hsa_circ_0005075	29710484	GO:0035198,GO:0010468,GO:0030334,GO:0030260,GO:0042127
hsa_circ_0005075	27258521	GO:0022407
hsa_circ_0005105	28276108	GO:0010628,GO:0090091,GO:0035198
hsa_circ_0005986	28410211	GO:0010629,GO:1900087,GO:0008284
hsa_circ_0006054	28484086	GO:0035198
hsa_circ_0006323	28947970	GO:0035198,GO:0036207
hsa_circ_0007874	28520103	GO:2000346,GO:0043066,GO:0035198,GO:0010628
hsa_circ_0011946	29593432	GO:0010468,GO:0030336
hsa_circ_0013339	30173892	GO:0030336,GO:0043065,GO:0030177

hsa_circ_0013339	28368401	GO:0010628,GO:0008284
hsa_circ_0016347	28424426	GO:0008284
hsa_circ_0018289	29156822	GO:0008285,GO:0030336,GO:0030260
hsa_circ_0020397	28707774	GO:0043066,GO:0030308,GO:0010628
hsa_circ_0022342	28682884	GO:0035198,GO:1901186
hsa_circ_0023404	29738762	GO:0008285,GO:0045786,GO:0030336
hsa_circ_0023404	26931159	GO:0090091,GO:0035198,GO:0010628
hsa_circ_0024169	25624062	GO:0008285
hsa_circ_0004712	28983619	GO:0035198,GO:0010468
hsa_circ_0032970	28947970	GO:0035198,GO:0036207
hsa_circ_0041103	27484176	GO:0010628,GO:0030335,GO:0008284,GO:0035198
hsa_circ_0043256	28958934	GO:0043065,GO:0008285
hsa_circ_0138960	28980874	GO:0008284,GO:0090091
hsa_circ_0051172	28947970	GO:0035198,GO:0036207
hsa_circ_0052012	28983619	GO:0035198,GO:0010468
hsa_circ_0054537	28947970	GO:0035198,GO:0036207
hsa_circ_0057576	28947970	GO:0035198,GO:0036207
hsa_circ_0064996	28983619	GO:0035198,GO:0010468
hsa_circ_0067934	27752108	GO:0030335,GO:0035197,GO:0008284,GO:0045785
hsa_circ_0068942	28947970	GO:0035198,GO:0036207
hsa_circ_0071410	28774651	GO:0035198,GO:2000491
hsa_circ_0082824	28947970	GO:0035198,GO:0036207
hsa_circ_0083357	28947970	GO:0035198,GO:0036207
hsa_circ_0083964	28983619	GO:0035198,GO:0010468
hsa_circ_0089378	28947970	GO:0035198,GO:0036207
hsa_circ_0047905	28980874	GO:0008284,GO:0090091
